# Anaerobic Digestion of Pretreated Industrial Residues and Their Energetic Process Integration

**DOI:** 10.3389/fbioe.2020.00487

**Published:** 2020-06-19

**Authors:** Günther Bochmann, Gunther Pesta, Lydia Rachbauer, Wolfgang Gabauer

**Affiliations:** ^1^Environmental Biotechnology, Department IFA-Tulln, University of Natural Resources and Life Sciences, Vienna, Austria; ^2^ATRES, Munich, Germany; ^3^Best Bioenergy and Sustainable Research, Tulln, Austria

**Keywords:** anaerobic digestion, food and beverage industry, residues, energy, brewery, slaughterhouse, bioethanol

## Abstract

The food and beverage industry offers a wide range of organic feedstocks for use in biogas production by means of anaerobic digestion (AD). Microorganisms convert organic compounds—solid, pasty, or liquid ones—within four steps to biogas mainly consisting of CH_4_ and CO_2_. Therefore, various conversion technologies are available with several examples worldwide to show for the successful implementation of biogas technologies on site. The food and beverage industry offer a huge potential for biogas technologies due to the sheer amount of process residues and their concurrent requirement for heat and power. The following study analyzes specific industries with respect to their implementation potential based on arising waste and heat and power demand. Due to their chemical composition, several feedstocks are resistant against microbiological degradation to a great extent. A combination of physical-, chemical-, and microbiological pretreatment are used to increase the biological availability of the feedstock. The following examples will discuss how to best implement AD technology in industrial processes. The brewery industry, dairy production, slaughterhouses, and sugar industry will serve as examples.

## Introduction

During several industrial processes, huge amounts of organic residues are produced which need to be treated or disposed of. This applies especially for the food and beverage industry. During food processing, liquid, solid, and pasty-like residues arise constantly or seasonally. At the same time, energy in the form of heat or power is required during the processing. The demand for power and heat depends on the production process, the region, and process technology. Several industrial processes such as brewing require saturated steam, while others require lower temperature levels. Breweries apply saturated steam for the brewing process and bottle washing and power for pumps, cooling, etc. (Muster-Slawitsch et al., 2011). Whereas, during the slaughtering process, lower temperature levels are needed. The dairy industry requires heat for raw milk treatment and cleaning, and, like all other industries, requires power for the production process itself (pumps, stirring, cooling, etc.) (Ortner et al., 2015b; Mainardis et al., 2019). The amount which is needed for each process depends on the industry, type of process, and amount of energy recovery by heat exchangers (Fritzson and Berntsson, 2006; Muster-Slawitsch et al., 2011; Ortner et al., 2015b). For all mentioned industries there is always a mixture of heat and power required. Thus, in many factories natural gas is already an important energy vector covering both heat and power. The production of biogas as a substitute to natural gas is an option to cover this energy requirement via the anaerobic degradation of inherent residues from the in-house production process (Pesta et al., 2006).

This paper gives an overview on four different residue intensive industries and the potential of using these residues for environmental process integration.

As mentioned, depending on the industry, huge amounts of residue occur and might also require a costly treatment. For breweries, this amounts to ~20 kg of fresh matter of brewers' spent grains and 300 l of wastewater per hl beer produced. When looking at the production of beer in Europe with a total of 412,221,000 hl in 2017, about 8.2 Mio t of brewers' spent grains occured (The Brewers of Europe, 2018). In 2012, the worldwide beer production was ~1,950 Mio hl (Alliance, 2014).

The consumption of meat and livestock products, like eggs and dairy, increases continually. This applies for all regions with differences in increase percentage. On average, the daily caloric intake from meat products was about 500–750 kcal per person in 2009 (Food and Agriculture Organization, 2009).

The numbers of livestock units increased tremendously during the period from 1987 to 2007 (Table 1). In 2007, the amount of meat production (beef, mutton, pork, and poultry meat) accounted for 241.5 Mio t. Although more recent numbers from the FAO are not yet available (Food and Agriculture Organization, 2009) the trend was ongoing within the past few years. Consequently, the amount of residue from these tremendous production volumes which require disposal is steadily increasing also.

**Table 1:** Production of livestock units developing status in Mio. Mg (Food and Agriculture Organization, 2009).

**Regions**	**Pig**		**Poultry**		**Cattle**		**Sheep and goat**	
	**1987**	**2007**	**1987**	**2007**	**1987**	**2007**	**1987**	**2007**
Developed countries	37.1	39.5	22.9	37.0	34.1	29.4	3.7	3.2
Developing countries	26.6	76.0	13.0	49.8	16.9	32.5	5.0	10.8
Total	63.6	115.5	35.9	86.8	50.9	61.9	8.6	14.0

At the beginning of this century, worldwide milk production was at 594.4 Mio m^3^ each year (Food and Agriculture Organization, 2019). These numbers further increased to over 840 Mio. m^3^ in 2018 (Food and Agriculture Organization, 2009). Each m^3^ of processed milk produces ~0.4–0.5 m^3^ of wastewater, which means a total amount of roughly 400 Mio m^3^ of wastewater each year. This wastewater needs to be treated due to its high nutrient content which would otherwise pollute the environment (Pesta et al., 2007).

Worldwide sugar production is based on sugar beet and sugar cane. In 2016, global sugar production reached 176.9 Mio t. 22.5% of sugar originating from sugar beet and the rest from sugar cane. Sugar cane is cultivated from sugar sources in southern regions. Sugar cane is cultivated in 104 countries, covering an area of 26 million hectares. Brazil is the largest producer with a volume of 35.48 million t of sugar, 28.16 billion l of ethanol, and 25,482 GWh of electricity (Grassi and Pereira, 2019). Sugar beet is cultivated in 54 countries with a quantity of 269.7 Mio t of raw sugar beet root. Europe is the largest producer with ~67% of the total amount of sugar beet root (Rajaeifar et al., 2019).

## Pretreatment Technologies

### Overview Pretreatment Technologies for AD

In the following section, several pretreatment technologies are described and their principles of function explained. Additionally, data are presented from several studies and their outcomes regarding the specific residue of the food and beverage industry are discussed.

Pretreatment technologies can be divided into a combination of physical, chemical, and biological technologies. Mechanical and thermal treatment includes physical pretreatment with knives, mills, ultra-sonic, or electrokinetic disintegration. Chemical pretreatments are defined as the addition of acids, bases, or various solvents to the feedstock in combination with a specific retention time. Biological pretreatment can be defined as pre-acidification, also called dark fermentation or hydrolysis, which is misleading because acidification takes place alongside hydrolysis. Combined processes include steam explosion, thermochemical pretreatment, and AFEX (ammonium fiber explosion). Depending on the feedstock and its chemical composition, specific pretreatment technologies are useful. In the following you will find a brief description of pretreatment principles and technologies.

### Physical Pretreatment

Thermal, mechanical, ultrasound, and electro kinetical disintegration are summarized as physical pretreatment technologies (Bochmann and Montgomery, 2013). Thermal pretreatment principles are with or without the addition of water and involve temperatures of up to 200°C or even more. Through the higher temperature, organic complexes swell and microorganisms during anaerobic digestion can more easily attach with their enzymes to the chemical compound and degrade the connection. Mechanical pretreatment involves treatment with knives, mills, etc. Through this treatment the particles are degraded and the surface area increases. This leads to a fast degradation of the organic compounds because of the increased surface area to attach to. Ultrasound and electro kinetical disintegrations are carried out with a probe and leads to the penetration and destruction of the microbiological cells. The probes do not degrade biomass actively but destruct microorganisms, which leads to the release of enzymes of the microorganisms (Bochmann, 2019).

### Chemical Pretreatment

Chemical pretreatment comprises all treatments where acid or alkaline solution, or ammonia or organic solvents are added, mostly in a separate preconditioning vessel. The chemicals crack chemical compounds of, for example, lignocellulosic compounds and increases the bioavailability toward microorganisms (Rabelo et al., 2011). After a defined exposure time the pretreated feedstock is fed to the biogas digester. This technology is often used for second-generation biofuels, like bioethanol from bagasse or straw with an additional enzyme dosage, to obtain readily available sugars for the subsequent fermentation step (Pereira et al., 2015).

### Biological Pretreatment

Biological pretreatment can be divided in two basic categories: (a) enzymatical and (b) microbiological pretreatment. Enzymatical pretreatment means the mere addition of enzymes or enzyme products without any active microbiology involved. In general, these enzymes are produced from fungi or bacteria in a completely decoupled previous fermentation (Bochmann et al., 2007; Prasad et al., 2019).

Microbiological pretreatment, also called dark fermentation, pre-acidification, or multi-phase digestion, is a simple technology for the preconditioning of feedstock. During this first stage, the hydrolysis and acidification step is separated from acetogenesis and methanogenesis. This does not mean a strict separation and that all feedstock completely hydrolyses during the pretreatment. During the pre-acidification the amount of liquid intermediates e.g., with fatty acids, alcohols, and gases, mainly CO_2_ and H_2_, is increased (Deublein and Steinhauser, 2010).

### Combined Processes

Under combined processes the combination of different pretreatment principles is summarized. This category comprises steam explosion, thermochemical processes, extruders, etc. Steam explosion combines two physical principles. First, a closed vessel with feedstock is heated up by steam, hot water, or via an external heating jacket. After a substrate specific hold time the pressure is released abruptly. During this pressure release the water evaporates and disrupts, thus processing the biomass through this explosive release (Ziegler-Devin et al., 2019). Thermochemical pretreatment describes a combination of temperature and chemicals addition like acids, alkaline solutions, ammonia, or organic solvents (Costa et al., 2014; Harris et al., 2017; Mokomele et al., 2018; Bochmann, 2019).

These processes are applied for hard to degrade substances such as bagasse rather than for easily available feedstocks such as whey. This is due to a relatively high energic input for the pretreatment procedure. The additional energy input hast to be compared to the expected increase in gas yield during anaerobic digestion to the pretreatment to balance economics.

## Anaerobic Digestion of Residues from Different Industries

### Breweries

During the brewing process several residues are produced that can be used for anaerobic digestion of the organic fraction. Beside BSG, cold and hot break, residual yeast, and wastewater also arise and need to be disposed of or treated. Table 2 gives an overview of the amounts of residue from the brewing process. Brewers' spent grains exhibit by far the biggest organic amount and thus, the largest biogas potential.

**Table 2:** Organic residues in breweries applicable for biogas production by anaerobic digestion (Pesta et al., 2006; Muster-Slawitsch et al., 2011).

**Residue**	**Amount**
Brewers spent grains	20–22 kg*hl^−1^
Yeast	2.0–2.6 kg*hl^−1^
Hot break	0.4–2.0 kg*hl^−1^
Cold break	0.1–0.3 kg*hl^−1^
Malt dust	0.05–0.25 kg*hl^−1^
Kieselguhr	0.4–1.1 kg*hl^−1^
Labels	0.29 kg*hl^−1^
Waste water	0.35–0.4 m3*hl^−1^

In general, BSG is used as animal feed but is stable only for a few days before it starts degrading. Reduced amounts of cattle, changes in feeding practice, and green thinking of breweries has led to the rise of the idea of anaerobic digestion of this specific residue (Bochmann et al., 2015).

Per 1 hl beer produced about 18–20 kg hl^−1^ of residue with a dry matter of about 20%. Large scale breweries produce several million hl each year on average. As an example, Gösser Brewery, one of the largest breweries in Austria, produces about 1 Mio. hl beer annually resulting in ~20,000 tons of brewers' spent grains each year (IEA Bioenergy Task 37, 2018). Various studies carried out biomethane potential analyses or continuous digestion trials on BSG and reported a biogas potential in the range of 270–380 l kg^−1^ substrate. However, without any co-digestion of further feedstock addition like manure, supplementation of trace elements is required to keep up a stable anaerobic digestion process (Vavilin et al., 2008; Bochmann et al., 2015; Bougrier et al., 2018; IEA Bioenergy Task 37, 2018).

#### Pretreatment of Feedstock From Breweries

Several studies investigated the effect of pretreatment on BSG to increase biogas yield by anaerobic digestion. The aim was to increase the degradation rate as well as the overall gas yield. Mechanical pretreatment was realized by Weger et al. (2017), and improved anaerobic digestion after a thermal pretreatment was reported by Bochmann et al. (2015). Depending on the temperature level, an increase of ~28% compared to untreated was achieved in continuous digestion trials (Bochmann et al., 2015). In contrast, an enzymatical pretreatment did not reach these higher yields when compared to the thermal treatment procedures (Bochmann et al., 2007). At a large- scale, anaerobic digestion of BSG as a main feedstock relies on the application of a microbiological pre-acidification. Hereby, volatile fatty acids are produced in a CSTR with a retention time of ~3 days. This pre-acidified substrate is then pumped into the main digester. Feeding regimes at this large scale usually apply intervals with regular feeding only from Mondays to Fridays (IEA Bioenergy Task 37, 2018).

#### Process Integration

Energy recovery within the brewing process is a highly relevant topic due to economic and ecologic reasons. Two methods are available to increase the overall energy efficiency of the brewing process. One option is the installation of the most efficient processing technologies for the energy intensive processes of mash pan or wort boiling. The other option is to carry out a pinch analysis to determine specific energy sinks and potential sources for energy recovery during the production process. Muster-Slawitsch et al. (2011) identified an average breweries' energy demand with about 37 MJ hl^−1^ beer (Muster-Slawitsch et al., 2011). Compared to the Austrian example of Gösser Brewery presented by Muster-Slawitsch et al. (2011), some other closely monitored and evaluated breweries have an up to 4 times higher energy demand during the brewing process (Kubule et al., 2016). Thus, the potential for energy recovery in many breweries is huge.

If we compare the energy demand to the energy potential that could unfold by utilizing the residues from the brewing process, the calculation on energy recovery can be done (Table 3). For wastewater, a COD (Chemical Oxygen Demand) of 3,000 mg l^−1^ and a degradation efficiency of 90% was assumed for calculation.

**Table 3:** Energy potential from different residues from breweries (Connaughton et al., 2006; Muster-Slawitsch et al., 2011; Bochmann et al., 2015).

**Substrate**	Amount kg FM*hl^**−1**^	**Methane potential**	Total methane L*hl^**−1**^	**Energy kWh/MJ**
BSG	22	75 L*kg^−1^ FM	1,640	16.4/59.2
Wastewater	300	0.76 L kg^−1^ L	227	2.3/8.2
			Total	18.7/67.4

Table 3 shows the calculation of the energy potential in the two main residual streams. Data from Muster-Slawitsch et al. (2011) and Kubule et al. (2016) clearly demonstrate that the implementation of anaerobe technology for residue degradation is technologically and economically feasible. Kubule et al. (2016) presented several breweries and their energy requirement during brewing operations. In order to allow energy recovery by AD integration, breweries require an energy efficiency analysis and have to establish activities to reduce the specific energy demand during the brewing process (Muster-Slawitsch et al., 2011; Kubule et al., 2016). If breweries increase their energy efficiency, anaerobic treatment of BSG and wastewater treatment to produce biogas is an alternative to enable the switch to a fossil-fuel-free brewing industry. Such efforts are well in line with the topic of Corporate Social Responsibility which reached the brewing industry several years ago. Since then, many breweries have already set up a plan to save energy, resources, and water (Kawa and Łuczyk, 2015).

### Slaughterhouses

Another food processing industry which requires a lot of energy on the one hand while producing residue which needs to be disposed of on the other hand are abattoirs. The main residues are wastewater and animal byproducts like blood, rumen content, fats, and stomach content. Most of these residues need to be treated before they can be released to the environment for final disposal (Ortner et al., 2015b). Residues from abattoirs can be divided into cow, swine, and poultry animal byproducts. Further residues from e.g., horse, sheep, goat, or fish produce will not be considered in this publication.

Anaerobic digestion of slaughterhouse residues is interesting for economic reasons but exposes several risks due to the high nitrogen level of several residues. In Europe, utilization in anaerobic digestion plants is restricted by the Animal Byproduct Regulation 1069/2009/EC. The regulation manages the legislation to use animal byproducts and to avoid the outbreak and spread of diseases like the Bovine Spongiform Encephalopathy (BSE) and the Creutzfeldt-Jakob disease, which is dangerous to humans (EC, 2015). The various byproducts are split into three categories. Category 1 consists of meat and animal byproducts with the highest risk from animals. These animals are killed or die due to disease, in particular BSE infested carcasses, or from contamination with chemicals or prohibited substances. Category 1 material must be incinerated and cannot be used for AD at all. Category 2 contains meat and by-products presenting a risk of other diseases. It includes killed and fallen, i.e., not slaughtered, animals, animal by-products (e.g., milk), imported, and insufficiently controlled material, animal products containing residues of medicines, and organs found to be infectious during the slaughtering process. Category 3 material mainly refers to waste and by-products from slaughterhouses, catering waste, food of animal origin no longer fit for human consumption, raw milk, fresh fish, or fresh fish by-products (EC, 2015).

The weight of residues per animal varies strongly and depends not only on the size of livestock but also on the region. Afazeli et al. (2014) presented cattle with 250 kg and poultry with 1.5 kg in an Iranian context, whereas Wang et al. (2018) presented data from the US with 618 and 2.8 kg, respectively. The amount of blood from cattle varies from 3.8 to 7%, and the rumen content from 10 to 15% (Afazeli et al., 2014; Wang et al., 2018). Thus, the region and specification of the animal needs to be considered when calculating amounts of by-products for biogas production. Ortner et al. (2014) presented the following data (Table 4).

**Table 4:** Gas potential, VS, and classification of residues from abattoirs (Ortner et al., 2014).

**Waste fraction**	**TS (%)**	**BMP (${\text{Nm}}_{\text{CH4}}^{\text{3}}$/Mg_**VS**_)**	**Category**
Blood	18–22	510–545	3
Stomach content	14–15	773–810	2
Grease separation	10–12	742–775	3
Rumen content	12–14	338–358	2
Waste water	0.8–1.2	746–852	

The challenge during anaerobic digestion is the high nitrogen content of several residues like blood. BMP potential tests show high gas yields but, during continuous digestion, inhibition of the methanogenic archaea, especially of acetoclastic methanogens, occurs (Moestedt et al., 2016). Depending on the ammonia level and the retention time residues can be digested completely. Still, several studies proved that nitrogen rich feedstock can be digested without huge process instabilities even on a long term (Ortner et al., 2014, 2015b). Specifically the addition of trace elements showed stabilizing properties during anaerobic digestion of nitrogen rich feedstock like blood and also during anaerobic wastewater treatment (Ortner et al., 2015a; Schmidt et al., 2018).

#### Pretreatment of Feedstock From Slaughterhouses

Depending on the feedstock and its regulatory requirements, animal byproducts need to be thermally treated prior to AD. Category 2 material may be transferred to a biogas plant after pressure sterilization (i.e., conditions as in an animal basket disposal plant: 50 mm, 133°C, 3 bar for 20 min). Category 3 material may be used in biogas plants after pre-crushing (12 mm) and pasteurization (70°C for 60 min). In general, pasteurization is carried out in batch processes, but some continuous systems are successfully in operation as of today. The benefits of a continuous system are lower operation costs, including energy, personal, and investment costs. Additionally, less components like a cooling tank, air scrubber, and various pumps are required (Wöss et al., 2019). It has to be stated that this pretreatment does not have any influence on the final biogas yield but enhances the degradation speed. In 2011, Rodríguez-Abalde et al. (2011) analyzed the influence of pasteurization on biomethane potential. The degradation time to reach the maximum yield was decreased by only 2–3 days. Luste and Luostarinen analyzed in 2010 an increased biogas production by pasteurization of 10 and 24% at retention times of 25 and 20 days during anaerobic digestion, respectively. However, in this study, sewage sludge was co-digested. It needs to be pointed out that the longer the retention time is, the smaller the additional gas yield is Luste and Luostarinen (2010) and Rodríguez-Abalde et al. (2011).

#### Process Integration

Some studies deal with the topic of integrating anaerobic digestion of animal by-products to cover the energy demand of an abattoir. Theoretical calculations of an Irish case were presented by Ware and Power (2016) with a focus on slaughterhouses dealing with cattle. It was demonstrated that 100% of the energy demand can be covered by biogas produced from the residues of slaughtering. Compared to the Irish energy demand, the utilization of all residues in various biogas plants could cover 1.63% of the energy demand of the whole Irish industry (Ware and Power, 2016). In 2015, Ortner et al. calculated a slaughterhouse and the self-sufficient energy supply by process integrated environmental protection. The calculation is based on data from a real biogas plant at a production site in Upper Austria. The annual energy demand lies between 5.5 and 7.0 GWh for electricity and 4.5–6.0 GWh of thermal energy. All in all, ~13,700 t of animal by-products occur each year and are treated via AD. This does not include bones, skins, heads, and eyes, which account for 3,500 t of the total amount of residue. The annual costs of this specific slaughterhouse for waste disposal without any treatment is in the range of 1.4 Mio €. This calculation is based on the estimated costs of about 15–50 € per t residue. The produced biogas is combusted in a CHP unit and covers 50% of the heat demand and 60% of the electricity demand of the slaughterhouse. Thus, the cost reduction for reduced external energy demand and avoided disposal cost by implementation of AD sums up to about 63% (Ortner et al., 2015b).

### Dairy

During milk processing in dairies, huge amounts of liquid residue, especially whey and wastewater, occur. Due to their chemical composition these residues need to be treated before being disposed of and released to the environment. High protein, phosphor, sugar, and salt content build a good basis for eutrophication of waters if the residues are released to nature untreated. Due to the vast number of dairies worldwide, proper treatment of residue is important for environmental protection (Escalante et al., 2018). Residue from milk processing industries can be divided into cheese whey, second cheese whey, and wastewater (Carvalho et al., 2013). Furthermore, the production of urda and ricotta from sweet whey is an opportunity to use proteins, mostly albumin, sugars, and fats (Paskaš et al., 2019). The characteristics of each liquid are presented in the following Table 5.

**Table 5:** Composition of effluents from dairies (Calli and Yukselen, 2002; Demirel et al., 2005; Panesar et al., 2007; Pesta et al., 2007; Chatzipaschali and Stamatis, 2012).

**Effluent**	**pH**	COD [mg*l−1]	TS [g*L−1]	VS [g*L−1]
Sweet whey	6–7	55,000–95,000	50–70	4.4–52.7
Acid whey	<5	63,100	63–70	
Wastewater	4–11	700–3,000	2.7–3.7	0.255–0.83

Because of the sheer amount of these liquid residues, they need a cost efficient and sustainable treatment. Economics strongly depend on the size of the dairy. While smaller plants require low cost solutions like utilization as animal feed, bigger plants can go far beyond that for the utilization of whey in the form of whey powder. Independent of size, anaerobic digestion is an interesting technology from small to large dairies due to economic and ecological reasons (Valta et al., 2017; Escalante et al., 2018; Mainardis et al., 2019). Several technologies have been developed and analyzed within the past few years to valorize whey and to produce an energy vector like biogas. Dairies benefit in two ways when treating the residues in this way. Firstly, it reduces the organic load of the effluent and secondly, additional energy is produced.

For the treatment of whey, various anaerobic technologies like CSTR (Continuous Stirred tank Reactor), EGSB (Expanded Granular Sludge Bed), UASB (Upflow Anaerobic Sludge Blanket), membrane reactor, hybrid systems, or combined systems with CSTR were determined (Ergüder et al., 2001; Calli and Yukselen, 2002; Saddoud et al., 2007; Diamantis and Aivasidis, 2018; Kumari et al., 2018; Ribera-Pi et al., 2018; Dereli et al., 2019; Ramos-Suárez et al., 2019). Additionally, aerobic systems are available. Many recent studies analyzed the performance of CSTR for whey treatment in co-digestion with feedstocks of high nitrogen or solid matter content (Martínez-Ruano et al., 2019; Ramos-Suárez et al., 2019). In general, wastewater from dairies is treated microbiologically using both aerobic and anaerobic systems. The different categories of wastewater are processing water, cleaning wastewater, and sanitary wastewater and show different chemical compositions and opportunities of utilization. Processing water can be reused after a minimal treatment due to its low pollution while cleaning and sanitary wastewater require extended treatment before disposal (Slavov, 2017). Applied technologies include UASB, Downflow-Upflow hybrid reactors, Anaerobic Moving-Bed Biofilm Reactors, Packed-Bed Immobilized Cell bioreactor, Downflow Stationary Fixed-Film reactor, and membrane bioreactors (Hassan and Nelson, 2012).

#### Pretreatment of Feedstock From Dairies

To enhance anaerobic digestion of dairy residue several pretreatment technologies were analyzed. Hidalgo et al. (2012) took a deeper look into mechanical and chemical pretreatment, as well as sonification for whey. The outcome was a marginal increase of digestibility by shredding whey. A larger increase in methane production of 10% was realized after sonification, and a negative influence was observed even after the addition of acids (H_2_SO_4_) and alkalines (NaOH) (Hidalgo et al., 2012). Another study analyzed ultra-sonic treatment with 20 kHz and 40 and 80 W combined at different retention times. Their findings revealed that a longer pretreatment leads to lower gas yields, whereas a lower energy input resulted in increased gas yields. Finally, pretreatment of liquid residues from dairies also led to a higher energy efficiency of the pretreatment compared to the energy yield after anaerobic digestion (Mainardis et al., 2019).

#### Process Integration

A techno-economic feasibility study on the integration of anaerobic digestion within dairies was carried out by Mainardis et al. (2019). Several different kinds of whey were analyzed for their biomethane potential. Additionally, the effect of ultra-sonic treatment on the digestibility of these whey streams was tested. As presented in the section pretreatment technologies, Mainardis et al. (2019) observed an increase in degradation rate and methane yield at low energy input. Based on these methane yields the energy demand of five selected small dairies were compared to the energy produced. For most dairies, biogas production covered the electrical and thermal energy requirements by more than 100% and thus, allowed for energy self-sufficiency. However, it was mentioned by the authors that the true energy demand seems to be higher based on the information given by the plant operators (Mainardis et al., 2019). In another study, the researchers calculated energy savings and revenues from struvite production for the use as fertilizer for dairies in Columbia. The economic benefit was US$ 6.91 and US$ 5.91 per m3 of whey for energy and struvite production, respectively (Escalante et al., 2018), with large-scale plants already in operation. One example from Austria has a modified floatation bed in operation since 2006. This dairy, which is located in Upper Austria, anaerobically treats 360 t d^−1^ acid whey and wastewater to produce 5,500 m3 biogas with a methane concentration of 58%. With an installed electrical capacity of 500 kW and almost the same thermal capacity, the daily energy production is 7,900 and 9,900 kWh for power and heat, respectively.

### Sugar Production

Sugar plays an important role in the food and beverage industry worldwide, and biofuel production is also strongly sugar based. The six biggest sugar producers are Brazil, India, Europe, Thailand, China, and the USA. These countries together produce more than 100 Mio t of sugar each year (Chunhawong et al., 2018). Sugar production relies on sugar cane and sugar beet as main crops with a global cultivable area of 6.232 Mio ha. Sugar cane and sugar beet hereby cover 1.762 and 4.47 Mio ha, respectively (Chunhawong et al., 2018; Rajaeifar et al., 2019). While in subtropical and tropical regions sugar originates from sugar cane, sugar is based on corn, wheat, or sugar beet in the rest of the world. Beside sugar as a main product, various residual streams still relatively rich in organics occur and can be utilized for purposes such as biogas production (Table 6).

**Table 6:** List of crops for sugar production.

**Crop**	**Product**	**Residue**	**References**
Sugar cane	Sugar	Bagasse, molasse, filter cake	Zang et al., 2018
Sugar beet	Sugar	Sugar beet pulp, molasse	Brooks et al., 2008

#### Sugar Cane

During the production of sugar from sugar cane bagasse, filter cake, and molasses accrue in large amounts. Utilization of bagasse is commonly used during biofuel production in the form of heat and electricity, and contributes to a certain extent to the energy production in smaller countries like Belize (Gongora and Villafranco, 2018). Filter cake can be fed to animals, and molasses (description can be found in the section sugar beet) is used in the food sector for alcohol production or as a food additive. Thus, in several countries sugar production is combined with alcohol production. Nevertheless, all these residues still contain significant amounts of organics and can also be used for biogas production during anaerobic digestion.

##### Anaerobic digestion of bagasse

In general, bagasse is used for heat production for e.g., downstream processing of sugar in combination with electricity generation, when needed (Gongora and Villafranco, 2018). Bagasse is the fiber of sugar cane, making up ~10–16% of the sugar cane plant, and consists of 39–46% of cellulose, 30–37% of hemicellulose, and 16–26% of indigestible lignin (Costa et al., 2014; Leite et al., 2015). Due to this composition, this feedstock shows a stubborn behavior during anaerobic digestion. For this reason, almost all pretreatment studies in the field of sugar-cane-derived residue deals with bagasse. Reports on biogas production from untreated bagasse show a huge difference from 35.6 to 229.6 l kg^−1^ VS. Some studies show a very short retention time of 30–33 days (Costa et al., 2014; Leite et al., 2015; Amin et al., 2017).

##### Digestion filter cake

During sugar production, filter cake represents another residue which is available for anaerobic digestion. Sugar cane is crushed, and sugar juice is extracted. Before processing to sugar, the sugar juice needs to be cleaned from impurities like dissolved and suspended solids by filtration. This filtrate is called filter cake or press mud (Kuruti et al., 2015). The biogas yield reported in literature for two different samples of filter cake within the same campaign showed a huge variety between 215 and 270 l kg^−1^ VS (Leite et al., 2015).

##### Vinasse digestion

Another potentially interesting residue for further utilization that is obtained after alcohol fermentation of molasses is vinasse. It is a liquid fraction after the distillation process containing organic compounds like residual sugars and volatile fatty acids (Leite et al., 2015). Vinasse is used in agriculture as a fertilizer or during anaerobic digestion to reduce COD. The COD load varies strongly and ranges between 16 and 100 g COD l^−1^ (Leite et al., 2015; López et al., 2018; Santana Junior et al., 2019). Anaerobic digestion trials of vinasse were carried out at lab scale, technical scale, and large scale in UASB, EGSB, and CSTR reactors as mesophilic or thermophilic digestion. BMP tests showed a potential biogas amount between 220.84 and 269.72 l kg^_COD_-1^ (Leite et al., 2015). COD degradation in UASB and EGSB was reported in a range of 60 to 80% (Leite et al., 2015; López et al., 2018).

##### Pretreatment of bagasse

A huge amount of bagasse occurs permanently during the separation of sugar juice from the sugar cane. Several studies worked on various pretreatment technologies of bagasse from sugar cane. Suggested pretreatment technologies include steam explosion, hydrothermal processing, microwave radiation, microbiological or enzymatic treatment, and chemical addition like alkali, acid, ammonia, or hydrogen peroxide (Costa et al., 2014; Leite et al., 2015; Bolado-Rodríguez et al., 2016; Amin et al., 2017; Mustafa et al., 2018). Physical pretreatment technologies especially resulted in an increase in biomethane production during anaerobic digestion of bagasse of 20% up to 900%. All these studies were carried out as batch trials with a short retention time of 30–35 days. Stubborn and hard degradable feedstock require a longer retention time to obtain a higher and more realistic gas potential. In case of comparing untreated and pre-treated hard degradable substrates, huge additional gas yields will be measured. The additional gas yield originates from an increased degradation rate and not from an increased yield (Hjorth et al., 2011).

##### Integration of anaerobic digestion into sugar plants

Opportunities and potentials of implementation for anaerobic digestion into sugar plants based on sugar cane are high. Beside bagasse, vinasse, and filter cake or press mud are utilizable for biogas production. It should be noted that digestion of bagasse competes with combustion and production of second-generation bioethanol from bagasse.

##### Integration of residues from sugar cane

Vinasse is a liquid formed from the fermentation of molasse and shows good fermentability during anaerobic digestion. This gas can be used in boilers to produce heat or heat and electricity by CHP units. It was calculated that biogas production and its utilization leads to 9–11% replacement of the total energy demand provided by bagasse combustion (data were only available from bioethanol plants based on sugar cane) (Nakashima and de Oliveira Junior, 2020). An alternative utilization of biogas was described by Leme and Seabra (2017). They compared the different systems for gas upgrading for biogas from vinasse. Biogas upgrading units separate CO_2_ from CH_4_. Biogas upgrading provides the energy vector biomethane, which is of equal quality to natural gas and can be used as a substitute of such. The upgrading technologies analyzed were pressure water scrubbing, organic-physical scrubbing, amine scrubbing, membrane separation, and pressure swing adsorption. The costs for such upgraded biogases to biomethane quality were between R$ 30 and 34 per GJ, which in Brazil is in the range of imported gas (Leme and Seabra, 2017).

Anaerobic digestion of bagasse requires a long retention time which means large digesters or the implementation of pretreatment technologies to increase the degradation rate and yield. Costa et al. (2014) compared anaerobic digestion of pretreated bagasse using alkaline, acid, and hydrothermal pretreatment technologies with direct combustion regarding the energy yield. Compared to combustion, anaerobic digestion showed a slightly lower energy yield of 0.1 MJ kg^−1^ dry bagasse (Costa et al., 2014).

##### Sugar beet

Twenty percent of the world's sugar production origins from sugar beet. The main producers of sugar beet are Europe and the USA. The residues from sugar beet processing are sugar beet pulp (SBP) and molasses which are obtained in large amounts (Suhartini et al., 2018; Schmid et al., 2019). Per ton of fresh sugar beet, 140 kg sugar, 300–350 kg SBP and 35 to 50 kg molasses arise (Stoyanova et al., 2014; Zieminski and Kowalska-Wentel, 2017; Schmid et al., 2019). The composition of molasses from sugar cane is presented in Table 7.

**Table 7:** Composition of molasses from sugar cane and sugar beet (with sugar and desugarized).

	**Unit**	**Sugar cane**	**Sugar beet (Schmid et al., 2019)**	**Desugarized sugar beet (Schmid et al., 2019)**
Dry matter	%	73.5–87.5	82.0	70.7–71.6
Sucrose	%	15.7–46.9	50.5	13.2–17.6
Ash	%	–	10.9	20.4–25.5
Nitrogen	%	0.25–1.5	1.8	1.8-2.1
Protein	%	–	11.0	11.4–13.0
Phosphorus	%	0.3–0.7	0.02	<0.02
Sodium	g*kg^−1^	–	13.4	25.2–26.2
Potassium	g*kg^−1^	19–54	32.8	50.3–74.2
Calcium	mg*kg^−1^	6–12	111	235–255

##### Utilization of molasses from sugar production

The production of sugar from sugar cane and sugar beet takes place in several steps and during both processes, molasses is obtained in large amounts with a high residual sugar content. Every ton of sugar produced requires a raw amount of 7 t of sugar beet with 0.25–0.35 t of molasses as a by-product (Schmid et al., 2019). The main utilization of molasses lies in the pharmaceutical and chemical industry, as animal feed or fermentation substrate for the production of baker's yeast, rum, or bioethanol (Eggleston and Lima, 2015; Matissek, 2016).

##### Anaerobic digestion of sugar beet pulp

Anaerobic digestion experiments at lab-scale as well as pilot-scale proved that sugar beet pulp is a suitable feedstock to produce biogas. Several studies carried out analysis in batch and continuous digestion. BMP (biomethane potential tests) of SBP show results of ~570 l biogas kg^−1^ (Suhartini et al., 2018). Comparing continuous thermophilic to mesophilic digestion of SBP, thermophilic digestion performed better, including higher specific methane production (of 24%) and unlikely foaming during anaerobic digestion. SBP show a high pectin content. Fast degradation of pectin reduces foaming during anaerobic digestion (Stoyanova et al., 2014). Improved dewaterability of digestate after thermophilic treatment was another important finding (Suhartini et al., 2014). Pectin content in SBP affects foaming due to a gelling agent.

##### Pretreatment of sugar beet pulp

To increase digestibility, several pretreatment technologies have been studied. Zieminski and Kowalska-Wentel (2017) compared the effect of enzymatical, mechanical, and thermal pretreatment and combinations of these on SBP. A huge increase of 96% in methane yield was reached by thermal pretreatment compared to untreated SBP, but the overall energy yield was lower due to the energy demand for pretreatment itself (Zieminski and Kowalska-Wentel, 2017). In another study, Zieminski et al. (2014) observed hydrothermal pretreatment on sugar beet pulp at temperature variations from 120 to 200°C in 20 K steps. The best results were realized at 160°C pretreatment temperature (Zieminski et al., 2014). It has to be mentioned that in both publications methane production and degredation degree from untreated SBP was very low compared to other publications, originating from a low retention time during the measurement of the methane potential (Hutnan et al., 2000; Brooks et al., 2008). Furthermore, microbiological pretreatment was analyzed by several authors. Depending on the process operation, SBP fermentation causes foaming. Stoyanova et al. (2014) analyzed the effect of microbiological pretreatment, in this case a pre-acidification of SBP, with regard to foaming. The study showed that this pretreatment strategy lowers the foaming effect during anaerobic digestion with lower retention time (Stoyanova et al., 2014).

##### Process integration

In 2008, Brooks et al. analyzed the implementation of an anaerobic digestion plant in a sugar beet processing unit. During the process campaign, 800 t SBP with 22% DM accumulated each day. In 2 cylindrical 12,000 m3 digesters the feedstock was digested within 30 days. The feed of SBP was reduced to 700 t each day and, at the same time, an additional 225 t of beet tails and leaves were fed. This resulted in an increased gas production of 125,000 m3 d^−1^ with 52 % methane. Biogas from anaerobic digestion of the residues covered 44% of the energy demand of the sugar beet processing plant (Brooks et al., 2008).

## Digestate Utilization

Anaerobic digestion produces renewable energy and recycles nutrients in the form of digestate. The liquid and solid digestate contains nutrients like nitrogen, phosphorus, and potassium, as well as humic compounds and microorganisms which positive affect soil quality when applied as fertilizer. Depending on national legislation, application of digestate is regulated and can be applied on fields. Artificial fertilizer will be substituted which increases the sustainability of the implementation of anaerobic digestion in the industry. Regulation implies the used feedstock, application sector, application time, and composition of the digestate (Al Seadi and Lukehurst, 2012; Tyagi et al., 2018).

During anaerobic digestion of residues from four different industries (brewery, slaughterhouse, dairy, and sugar industry) that are analyzed in this publication, digestate with divers characteristics is produced. The composition of nutrients depends on the input materials and the installed fermentation technology (Table 8).

**Table 8:** Composition of the digestate of the four different industries.

**Parameters**	**Units**	**Brewery**	**Slaughterhouses**	**Dairy**	**Sugar**	
Feedstock		BSG*	Blood, rumen content, fats*	Cheese whey wastewater (Ergüder et al., 2001)	SBP (Suhartini et al., 2018)	Bagasse (Janke et al., 2016)**
TS	g*kg^−1^	21.3	5.88	n.a.	–	433
VS	g*kg^−1^	15.6	5.13	n.a.	–	418
COD	mg COD*l^−1^	–	–	2,218–4,438	–	–
TKN	g*kg^−1^	5.54	7.3	0.15	4.04	1.24
Ammonia	g*kg^−1^	4.84	6.11	n.a.	1.40	n.a.
Phosphorus	g*kg^−1^	0.43	0.67	0.12	0.61	0.1
Potassium	g*kg^−1^	0.56	0.17	n.a.	0.72	

* *Monitoring campaign from institute. Unpublished data*.

** *Data based on input material*.

*n.a. = not available*.

All studies about digestion of bagasse were carried out in co-digestion trials. The numbers for bagasse are calculated based on data from literature including TS, gas production, and methane concentration.

Through the composition of digestate, biogas plant operators produce a valuable product. In 2018, Havukainen et al. calculated for each kg of mineral fertilizer nitrogen and phosphorus, 1.9–7.8 kg_CO2eq_ and 2.3–4.5 kg_CO2eq_, respectively. Comparing GHG emissions of organic fertilizer to mineral fertilizer, an average reduction of 78% for nitrogen and 41% for phosphorus occurs (Havukainen et al., 2018).

## Conclusion

The presented biomass processing industries (breweries, slaughterhouses, dairy production, and sugar processing plants) show a huge number and vast quantity of organic residues which occur continuously. All discussed waste streams can be treated anaerobically and are well-suitable for biogas production. For several of these residues, competitive utilization opportunities like animal feeding (e.g., brewers spent grains and, in some countries, dried blood) or for use in energy production (e.g., bagasse) do exist. But the majority of the residues are without any competitive or alternative use. One strength of anaerobic digestion is the opportunity to treat different residues within one single process, e.g., liquid and paste-like substrates. Thus, a company can implement one plant to treat several of their residues at once. The production of energy from residue offers the company the opportunity of energy independency. To reach energy autarky, companies need to increase the energy efficiency during the industrial process. In addition to the energy production, fertilizer production helps to reduce CO_2_ equivalents of the process.

This process integrated help from environmental protection to implement a Corporate Social Responsibility (CSR) strategy. Such CSR strategies are used for marketing and public relation departments in companies and support the green labeling of companies.

## Author Contributions

GP: the field of dairy and brewery. LR: slaughterhouse. WG: process integration. GB: pretreatment, bioethanol, and brewery. GB, GP, LR, and WG contributed to the work in his field of expertise.

## Conflict of Interest

GP is the owner of the company ATRES. The remaining authors declare that the research was conducted in the absence of any commercial or financial relationships that could be construed as a potential conflict of interest.
